# Temporal Trends in the Prescription of Biosimilars and the Status of Switching from Original Biologics to Biosimilars at Individual and Institutional Levels in Japan

**DOI:** 10.1007/s43441-025-00850-7

**Published:** 2025-08-07

**Authors:** Minako Matsumoto, Ryosuke Kumazawa, Akiko Ishii-Watabe, Itsuko Horiguchi, Hiroaki Mamiya, Hiroko Shibata, Yoshiro Saito, Motohiko Adomi, Yuta Taniguchi, Jun Komiyama, Ryoko Sakai, Masao Iwagami

**Affiliations:** 1 Department of Preventive Medicine and Public Health, School of Medicine, Keio University, Tokyo, Japan; 2 Department of Public Health and Epidemiology, Meiji Pharmaceutical University, Tokyo, Japan; 3 Division of Biological Chemistry and Biologicals, National Institute of Health Sciences, Kanagawa, Japan; 4 College of Pharmaceutical Sciences, Ritsumeikan University, Kyoto, Japan; 5 National Institute of Health Sciences, Kanagawa, Japan; 6 Department of Epidemiology, Harvard T. H. Chan School of Public Health, Boston, MA USA; 7 Department of Health Services Research, Institute of Medicine, University of Tsukuba, Ibaraki, Japan; 8 Health Services Research and Development Center, University of Tsukuba, Ibaraki, Japan; 9 Department of Digital Health, Institute of Medicine, University of Tsukuba, 1-1-1 Tennodai, Tsukuba, Ibaraki 305-8575 Japan; 10 Digital Society Division, Center for Cyber Medicine Research, University of Tsukuba, Ibaraki, Japan; 11 Department of Non-Communicable Disease Epidemiology, Faculty of Epidemiology and Population Health, London School of Hygiene and Tropical Medicine, London, UK

**Keywords:** Biological products, Biosimilar pharmaceuticals, Pharmacoepidemiology, Drug utilization, Administrative claims

## Abstract

**Purpose:**

To describe the temporal trends in the prescription of biologics in Japan, with additional analysis focusing on switching from original biologics to biosimilars at the individual and institutional levels.

**Methods:**

Using the JMDC claims database from January 2005 to May 2024, we identified patients who received at least one prescription for 17 biologics (original biologics or biosimilars). We elucidated the monthly trends in the proportions of original biologics and biosimilars. We also estimated the proportion of patients receiving original biologics only, those receiving biosimilars only, and those switching from original biologics to biosimilars (and vice versa) during the study period. Finally, we estimated the proportion of medical institutions that started prescribing biosimilars during the study period based on the type of medical institution.

**Results:**

Temporal trends in the proportions of original biologics and biosimilars varied widely. In May 2024, the proportion of biosimilar prescriptions was 13.6% for somatropin and 92.5% for filgrastim. At the individual level, the proportion of patients switching from original biologics to biosimilars was low (1.2–14.0%), indicating that switches do not often occur within the same patient, while more recent new users of biologics start biosimilars. At the institutional level, university-related hospitals and clinics were more and less likely, respectively to introduce biosimilars than public and other types of hospitals.

**Conclusion:**

Temporal trends in the prescription of biosimilars and switching patterns varied widely by the type of biologics. The type of medical institution should be considered when assessing and promoting the use of biosimilars. Further research and strategies to increase the use of biosimilars in clinics may be needed.

**Supplementary Information:**

The online version contains supplementary material available at 10.1007/s43441-025-00850-7.

## Introduction

Increasing medical costs has become a global social concern. Prescribed drugs, which account for a considerable part of medical costs, have received attention for potentially reducing its costs [1]. Generic drugs play a pivotal role in reducing drug costs and easing drug budgets [2].

Biologics currently occupy the top positions in drug sales [3]. Biologics are large and complex molecules with structural heterogeneity that have been developed for managing various diseases, including rare diseases [4]. Recently, a group of biologics known as “biosimilars” have been developed to potentially replace high-cost biologics [5]. Biosimilars are biotechnological products that are expected to be comparable to an already approved biotechnological product (referred to as “original biologics”) in terms of quality, efficacy, and safety [6].

Although the global market share of biosimilars is steadily increasing with efforts of governments and industries [7, 8], biosimilars have not fully penetrated the biologics market, probably due to healthcare professionals and patients concerns regarding their real-world effectiveness and safety [9, 10]. A previous systematic review targeting the US and Europe reported that the acceptance of biosimilars varied according to the therapeutic classes [11]. In Japan, although the adoption rate of some biosimilars such as erythropoietin, rituximab, or insulin glargine exceeds 60%, and that of filgrastim exceeds 90%, the majority remain at or below an adoption rate of 40% [12]. In a Japanese study that surveyed the adoption and prescription of biosimilars in four areas (hematology, medical oncology, rheumatoid arthritis, and inflammatory bowel disease), more than 60% of physicians expressed concerns about prescribing biosimilars, particularly regarding their efficacy, safety, and quality compared to original biologics [13].

Understanding the trends in the prescription of original biologics and biosimilars is crucial and can be considered the first step towards increasing the use of biosimilars worldwide, including Asia. In addition, researchers and policymakers should understand switches from original biologics to biosimilars at the individual and institutional levels. Replacement of certain original biologics by biosimilars in certain situations or settings, suggesting potential areas for improvement. To our knowledge, no Asian study has systematically assessed the temporal trends and switching patterns from original biologics to biosimilars, covering a variety of biologics [14].

In the present study, we aimed to describe the temporal trends in the prescription of original biologics and biosimilars in Japan, and performed an additional analysis focusing on switching from original biologics to biosimilars at individual and institutional levels.

## Methods

### Data Source

We used data from the JMDC claims database, which has been previously described in detail [15]. Briefly, the JMDC claims database is a large-scale database containing medical claims of large- and medium-sized company employees and their dependent family members aged < 75 years. Since 2005, the number of individuals in the JMDC database has increased consistently, reaching a cumulative total of more than 20 million by the end of 2024. The JMDC database includes all the monthly claims for outpatient and inpatient diagnoses and procedures, prescriptions, and dispensations of drugs recorded as the Japanese original drug codes and product names, and the World Health Organization Anatomical Therapeutic Chemical (ATC) classification [16]. In addition, the data included the anonymized IDs of medical institutions, with which we could discern which drug was prescribed by the medical institution, as well as the type of medical institution: clinics (defined in Japan as medical institutions with no or < 20 beds for hospitalization), university-related hospitals, public hospitals, and other hospitals (including private hospitals receiving reimbursement from the health insurance system in Japan). We employed the most recent dataset, extracted in November 2024, which included data from January 2005 to May 2024. The data used in this study were anonymized and processed anonymously by JMDC, Inc.

For comparison, we also used the National Database (NDB) Open Data of Health Insurance Claims from April 2022 to March 2023, which is a summary table of the total use of drugs compiled by the government [17] and covers all Japanese citizens except those living with public financial assistance.

This study was approved by the Ethics Committees of the University of Tsukuba, Ibaraki, Japan (approval number 2099) and Meiji Pharmaceutical University, Tokyo, Japan (approval number 202462). The analyses were conducted independently at each study location to verify and obtain similar results.

### Study Population

We identified patients receiving at least one prescription of all 17 biologics (for which biosimilars were approved and marketed by 2024) available in Japan during the study period, including somatropin, erythropoietin (including both epoetin alfa and epoetin beta), filgrastim, infliximab, insulin glargine, rituximab, etanercept, trastuzumab, agalsidase beta, bevacizumab, darbepoetin alfa, teriparatide, insulin lispro, adalimumab, insulin aspart, ranibizumab, and pegfilgrastim. For each of the 17 biologics, we created a list of product names (based on at least one prescription record in the JMDC database during the study period) using the ATC classification system, classifying them as original biologics or biosimilars (Supplementary Table S1). In the main analysis, we included all these biologics for our analysis. However, for several biologics (somatropin, erythropoietin, insulin glargine, darbepoetin alfa, insulin lispro, and insulin aspart), the original biologics other than the reference product of the biosimilar were approved (Supplementary Table S1). Thus, in the sensitivity analysis, we restricted the analysis to biosimilars and their reference products. In addition, there is one authorized generic (AG) biologic drug for darbepoetin alfa in Japan, which does not have a brand name on its label but is composed of the same drug component as the original biologics [18]. We included this AG biological drug in the biosimilars in the main analysis, but excluded it from our sensitivity analysis.

### Data Analysis

After summarizing the demographics (age and sex) of the study population by biologics, we estimated and illustrated the monthly trends in the proportion of prescriptions of original biologics and biosimilars (among the total prescriptions for each biologic) from January 2005 to May 2024. In addition, we compared the statistics (i.e., the number and proportion of biosimilars among the total prescriptions for each biologic) in the JMDC database with those estimated from the NDB Open Data of Health Insurance Claims from April 2022 to March 2023 [17].

Next, at the individual level, we estimated the proportions of (i) patients receiving original biologics only, (ii) those receiving biosimilars only, (iii) those switching from original biologics to biosimilars, (iv) those switching from biosimilars to original biologics, and (v) unknown (because both original biologics and biosimilars were prescribed in the same month, we could not determine which was prescribed earlier from the monthly claims alone) during the study period from January 2005 to May 2024 in the main analysis. Additionally, we restricted the period of the analysis from the time each biosimilar entered the Japanese market to May 2024.

Finally, at the institutional level, among medical institutions with at least one prescription of original biologics, we estimated the proportion of medical institutions starting the prescription of biosimilars during the study period by the type of medical institution (clinics, university-related hospitals, public hospitals, and other hospitals) and 17 biologics.

An additional analysis was carried out restricted to patients who visit medical institutions introducing biosimilars during the study period. In the same way as the main analysis, the proportions of (i) patients receiving original biologics only, (ii) those receiving biosimilars only, (iii) those switching from original biologics to biosimilars, (iv) those switching from biosimilars to original biologics, and (v) unknown, during the study period (from January 2005 to May 2024) and from the time each biosimilar entered the Japanese market to May 2024 were estimated.

All the analyses were performed using STATA version 17 software (StataCorp, College Station, TX, USA).

## Results

The number of study patients and their demographics varied by biologics, from 102 for agalsidase beta (mean age 38.3 ± 15.4 years, male 56.9%) to 62,038 for insulin glargine (mean age 52.0 ± 12.8 years, male 66.6%) (Table 1). In terms of the total number of prescriptions, insulin was most commonly prescribed (insulin glargine, 1,340,426 times; aspart, 1,173,924 times; and lispro, 1,151,886 times), followed by filgrastim (306,931 times). In the sensitivity analyses restricted to biosimilars (excluding AG biologic drugs) and their reference products, the total number of prescriptions decreased, especially for somatropin and darbepoetin alfa.

**Table 1:** Characteristics of studied drugs and study participants.

Name	ATC code	When the original drug was approved in Japan	When the biosimilar was approved in Japan	Total no. of prescriptions during the study period**	No. of patients with ≥ 1 prescription	Age*, mean ± standard deviation	Sex: no. of male patients (%)
(1) Somatropin	H01AC01	Nov, 1988	Jun, 2009	277,238	11,264	12.4 ± 11.3	6557 (58.2)
Sensitivity analysis***				80,791	3709	11.2 ± 8.9	2185 (58.9)
(2) Erythropoietin	B03XA01	Jan, 1990	Jan, 2010	159,167	20,175	30.7 ± 27.9	8754 (43.4)
Sensitivity analysis***				112,325	14,344	27.1 ± 28.1	6312 (44.0)
(3) Filgrastim	L03AA02	Oct, 1991	Nov, 2012	306,931	25,727	52.1 ± 15.6	12,627 (49.1)
(4) Infliximab	L04AB02	Jan, 2002	Jul, 2014	198,800	10,136	37.3 ± 15.8	6400 (63.1)
(5) Insulin glargine	A10AE04	Oct, 2003	Dec, 2014	1,340,426	62,038	52.0 ± 12.8	41,289 (66.6)
Sensitivity analysis***				1,017,736	51,381	52.3 ± 12.6	34,487 (67.1)
(6) Rituximab	L01FA01	Jun, 2001	Sep, 2017	87,361	8252	51.5 ± 16.2	4868 (59.0)
(7) Etanercept	L04AB01	Jan, 2005	Jan, 2018	192,029	7521	49.2 ± 12.0	1203 (16.0)
(8) Trastuzumab	L01FD01	Apr, 2001	Mar, 2018	227,537	9136	52.4 ± 9.2	523 (5.7)
(9) Agalsidase beta	A16AB04	Jan, 2004	Sep, 2018	11,869	102	38.3 ± 15.4	58 (56.9)
(10) Bevacizumab	L01FG01	Apr, 2007	Jun, 2019	268,449	14,261	55.0 ± 10.6	5691 (39.9)
(11) Darbepoetin alfa	B03XA02	Apr, 2007	Sep, 2019	131,241	13,083	55.2 ± 12.6	8480 (64.8)
Sensitivity analysis***				81,572	8491	55.0 ± 12.6	5440 (64.1)
(12) Teriparatide	H05AA02	Jul, 2010	Sep, 2019	74,155	5345	62.3 ± 8.6	938 (17.6)
(13) Insulin lispro	A10AB04 A10AC04 A10AD04	Aug, 2001	Mar, 2020	1,151,886	55,511	49.8 ± 13.9	32,012 (57.7)
Sensitivity analysis***				932,485	48,537	49.6 ± 14.0	27,663 (57.0)
(14) Adalimumab	L04AB04	Apr, 2008	Jun, 2020	196,998	10,558	40.3 ± 15.1	5655 (53.6)
(15) Insulin aspart	A10AB05 A10AD05	Apr, 2008	Mar, 2021	1,173,924	49,859	49.7 ± 14.7	28,693 (57.6)
Sensitivity analysis***				971,582	44,243	49.3 ± 14.8	25,123 (56.8)
(16) Ranibizumab	S01LA04	Jan, 2009	Sep, 2021	23,925	8160	56.0 ± 12.8	4846 (59.4)
(17) Pegfilgrastim	L03AA13	Sep, 2014	Sep, 2023	93,019	19,921	53.0 ± 11.0	5367 (26.9)

*Age at the time of first prescription in the JMDC claims database.

**From January 2005 to May 2024.

***While all the original biologics or biosimilars available in Japan were included in the main analysis, the sensitivity analysis made the changes below (corresponding to Supplementary Table S1).

For somatropin, we restricted to Genotropin vs. Somatropin BS.

For erythropoietin, we restricted to Espo vs. Epoetin Alfa BS (Epoetin Kappa).

For insulin glargine, we restricted to Lantus (not including Lantus XR) vs. Insulin Glargine BS.

For darbepoetin alfa, we restricted to Nesp vs. Darbepoetin Alfa BS (not including Darbepoetin Alfa authorized generic).

For insulin lispro, we restricted to Humalog (not including Humalog Mix and Humalog N) vs. Insulin Lispro BS.

For insulin aspart, we restricted to NovoRapid (not including NovoRapid Mix) vs. Insulin Aspart BS

**Figure 1:**
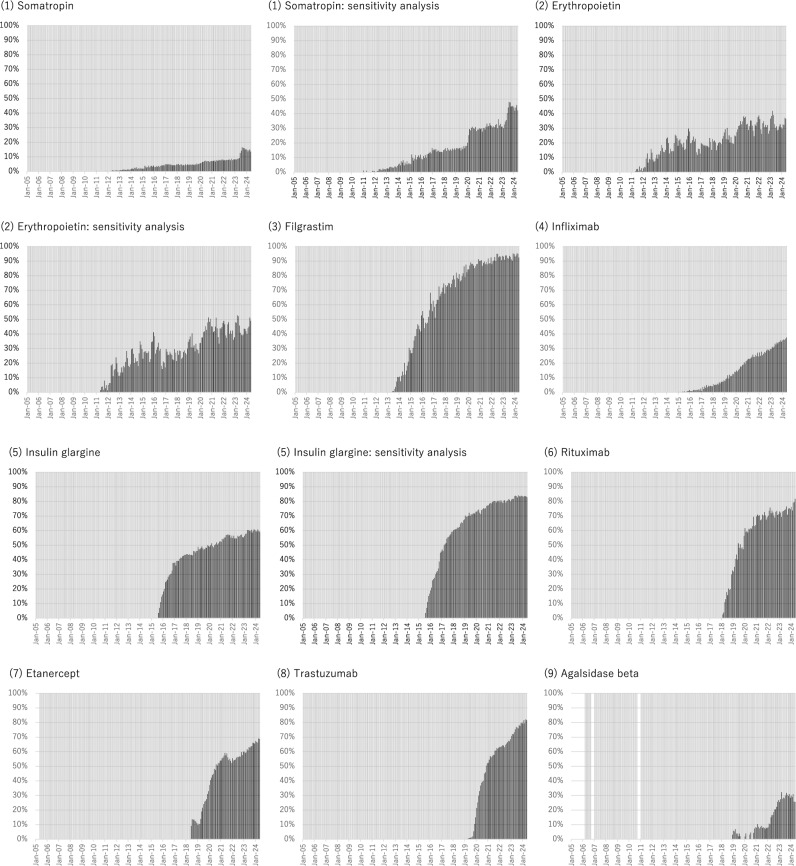

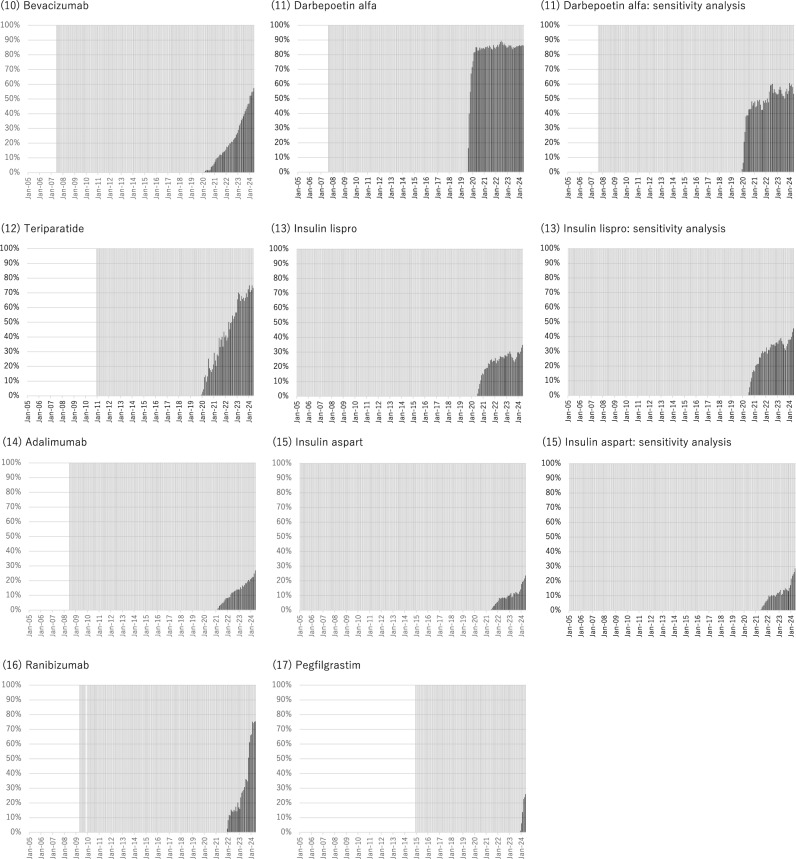
Monthly trend in the proportion of prescriptions of original biologics (colored gray) or biosimilars (colored black) among the total prescriptions for each biologic during the study period. Although all original biologics or biosimilars available in Japan were included in the main analysis, the sensitivity analysis made the following changes (Supplementary Table S1): For somatropin, we considered Genotropin vs. Somatropin BS. For erythropoietin, we considered Espo vs. Epoetin Alfa BS (Epoetin Kappa). For insulin glargine, we considered Lantus (not including Lantus XR) vs. Insulin Glargine BS. For darbepoetin alfa, we considered Nesp vs. Darbepoetin Alfa BS (not including Darbepoetin Alfa authorized generic). For insulin lispro, we considered Humalog (not including Humalog Mix and Humalog N) vs. Insulin Lispro BS. For insulin aspart, we considered NovoRapid (not including NovoRapid Mix) vs. Insulin Aspart BS.

The monthly trends in the proportion of original biologics and biosimilars varied widely among biologics (Fig. 1). Some biologics, such as filgrastim and trastuzumab, demonstrated a steep increase in the proportion of biosimilars since their launch, whereas biologics, such as somatropin and infliximab, demonstrated a slow increase. Darbepoetin alpha and insulin lispro initially demonstrated a steep increase, which became nearly flat. In May 2024, the proportion of biosimilar prescriptions (among all prescriptions of biologics) ranged from 13.6% for somatropin to 92.5% for filgrastim, as summarized according to the ATC classification of biologics (**Supplementary Table S2**).

The statistics in the JMDC claims database and the NDB Open Data from April 2022 to March 2023 were mostly similar (**Supplementary Table S3**), suggesting the generalizability of the findings in the present study.

**Figure 2:**
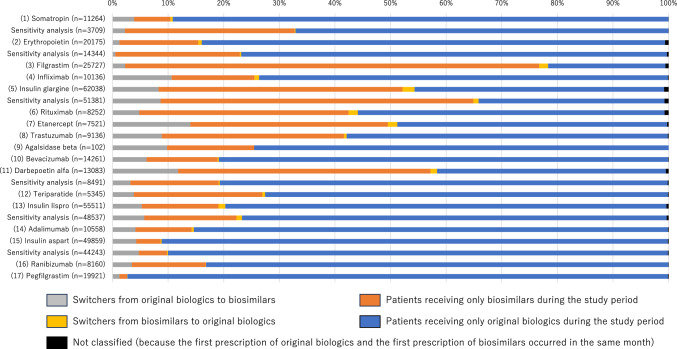
Distribution of patients receiving only original biologics or biosimilars during the study period or switchers. For switchers, only the first switch was assessed and counted (i.e., some patients switched twice or more). Although all original biologics or biosimilars available in Japan were included in the main analysis, the sensitivity analysis made the following changes (Supplementary Table S1): For somatropin, we considered Genotropin vs. Somatropin BS. For erythropoietin, we considered Espo vs. Epoetin Alfa BS (Epoetin Kappa). For insulin glargine, we considered Lantus (not including Lantus XR) vs. Insulin Glargine BS. For darbepoetin alfa, we considered Nesp vs. Darbepoetin Alfa BS (not including Darbepoetin Alfa authorized generic). For insulin lispro, we considered Humalog (not including Humalog Mix and Humalog N) vs. Insulin Lispro BS. For insulin aspart, we considered NovoRapid (not including NovoRapid Mix) vs. Insulin Aspart BS.

**Supplementary Table S4** and Fig. 2 illustrate the distribution of patients receiving original biologics only, biosimilars only, and those switching from original biologics to biosimilars, or vice versa. The proportion of patients switching from the original biologics to biosimilars was generally low, varying from 1.2% for erythropoietin to 14.0% for etanercept. The proportion of patients receiving biosimilars only was much higher than that of the patients switching for all biologics, implying that switches do not often occur within the same patient, whereas more recent new users of biologics start with biosimilars. The proportion of patients receiving biosimilars only was the highest for filgrastim (74.4%), followed by darbepoetin alfa (45.4%) and insulin glargine (43.9%). In the additional analysis, restricting the analysis period from when each biologic was launched (to May 2024), the proportion of switchers from the original biologics to biosimilars did not change significantly and remained low, whereas the proportion of patients receiving only biosimilars tended to increase (**Supplementary Table S4**).

**Figure 3:**
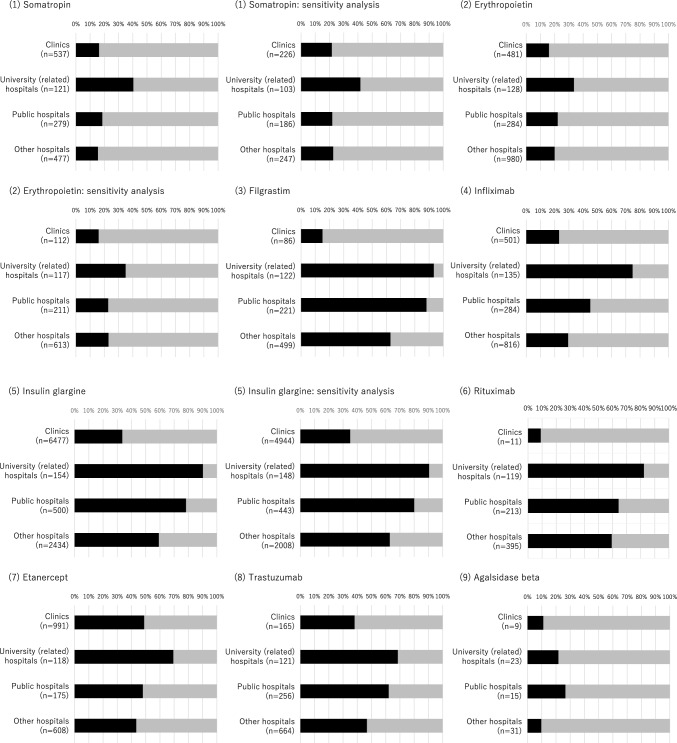

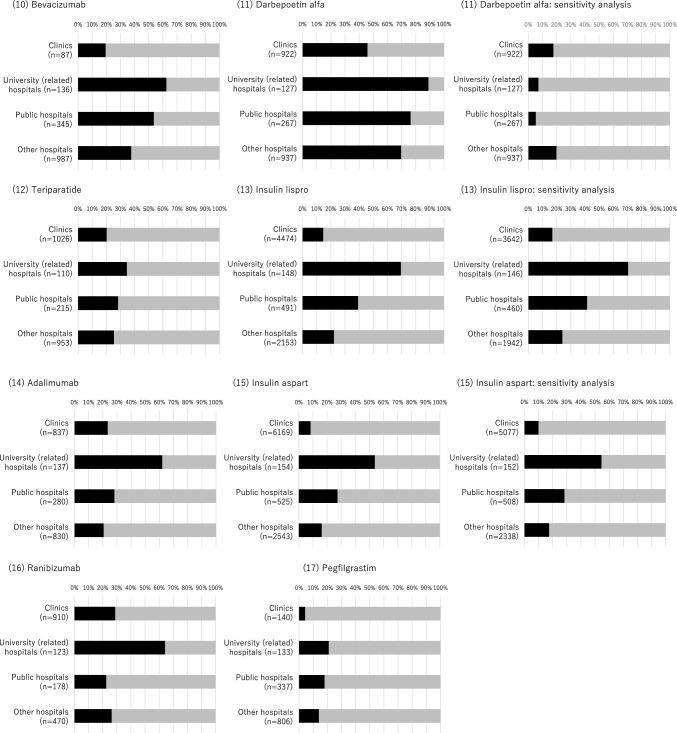
Distribution of medical institutions prescribing only original biologics (colored gray) or original biologics and biosimilars (colored black) during the study period. Although all original biologics or biosimilars available in Japan were included in the main analysis, the sensitivity analysis made the following changes (Supplementary Table S1): For somatropin, we considered Genotropin vs. Somatropin BS. For erythropoietin, we considered Espo vs. Epoetin Alfa BS (Epoetin Kappa). For insulin glargine, we considered Lantus (not including Lantus XR) vs. Insulin Glargine BS. For darbepoetin alfa, we considered Nesp vs. Darbepoetin Alfa BS (not including Darbepoetin Alfa authorized generic). For insulin lispro, we considered Humalog (not including Humalog Mix and Humalog N) vs. Insulin Lispro BS. For insulin aspart, we considered NovoRapid (not including NovoRapid Mix) vs. Insulin Aspart BS.

Finally, at the institutional level, for most biologics, the proportion of medical institutions introducing biosimilars during the study period (among those once prescribing original biologics as the denominator) was the highest in university-related hospitals, followed by public hospitals, other hospitals, and clinics (Fig. 3). However, insulin glargine and darbepoetin alfa were relatively commonly prescribed in all types of medical institutions compared with other biologics.

Regarding the analysis restricted to patients who visit medical institutions introducing biosimilars during the study period (**Supplementary Table S5**), the proportion of patients switching from the original biologics to biosimilars increased from those in our original analysis (**Supplementary Table S4** and Fig. 2**)** but was not markedly different. Again, the proportion of patients receiving biosimilars only was much higher than that of patients switching for all biologics, implying that switches do not often occur within the same patient, whereas new users of biologics start their treatment with biosimilars.

## Discussion

This study comprehensively investigated the temporal trends in the prescription of biosimilars, with an additional analysis focusing on switching from the original biologics to biosimilars at the individual and institutional levels, using the JMDC claims database for company employees and their dependent family members. The introduction of biosimilars varied widely by the type of biologics as well as by the type of medical institution. Switching within the same individual was uncommon, whereas more recent new biologics users started using biosimilars only. At the institutional level, university-related hospitals were more likely, and clinics were less likely, to introduce biosimilars compared to public and other types of hospitals.

The most frequently prescribed biologics were insulin (glargine, aspart, and lispro) merely because diabetes is a common disease, with an estimated 11 million patients in Japan in 2021 [19]. The number of prescriptions of insulin and filgrastim was proportional to the number of patients, while some biologics, such as erythropoietin and pegfilgrastim, were not, suggesting that the frequency and duration of use in individuals vary according to the biologics and related underlying diseases. Restricting our analysis to biosimilars (excluding AG biological drugs) and their reference products, the levels of somatropin and darbepoetin alfa decreased. This suggests that most of the patients treated with somatropin used the original biologics, which were not the reference products, whereas most of the patients treated with darbepoetin alfa used AG biologic drugs.

The monthly trends in the proportion of biosimilars varied widely according to the biologics. The use of biosimilars filgrastim, rituximab, trastuzumab, ranibizumab, and teriparatide demonstrated rapid increase during the study period. We did not find consistent characteristics that were obviously different between biologics with and without a rapid increase in the proportion of biosimilars, such as the timing of approval and underlying diseases. For example, biosimilars used in cancer treatment, such as filgrastim, rituximab, and trastuzumab, have demonstrated a rapid increase in use; however, bevacizumab (also used in cancer treatment) did not demonstrate a similar trend. One possible pattern may be that biologics used as a secondary or additional treatment, such as filgrastim (which is used for the prevention of neutropenia due to cancer chemotherapy), could be easier to replace with biosimilars than those used as a primary treatment.

We found some temporal stagnation or a decrease in the proportion of several biologics, such as etanercept, darbepoetin alfa, and filgrastim. One possible explanation is that some practitioners may have stopped using biosimilars owing to a shortage of biosimilars in Japan. Drug shortages occurred for etanercept in 2018 and 2021, darbepoetin alfa in 2020, filgrastim in 2020 and 2022, and pegfilgrastim in 2024. One of the major reasons for drug shortages is that the demand exceeds expectations. A stable supply of biosimilars is necessary to promote their use.

In addition, changes in the healthcare system or policy may have contributed to the increase in the use of biosimilars. In Japan, the revision of medical fees in 2020 explicitly promoted this use, creating financial incentives for healthcare institutions. The 2024 revision of medical fees expanded the range of targeted biosimilars compared to that from 2020, and the incentive scheme shifted from focusing on individual prescriptions to promoting institution-wide implementation of the use of biosimilars.

The proportion of switchers from original biologics to biosimilars was generally small. One obvious reason for the low switching proportion within the same individual is that switching from an original biologic to a biosimilar over the course of treatment was not recommended in Japan until 2022 to ensure the traceability of the products as per domestic guidelines. In addition, a previous study reported that some physicians [20] and patients [21] may be concerned about the safety profiles of biosimilars, especially when they switch from the original biologics to biosimilars, which can lead to negative expectations and nocebo effects [22]. A better understanding of biosimilars could help encourage their acceptance by prescribers and patients.

The introduction of biosimilars varies according to the type of medical institution. The proportion of clinics that introduced biosimilars was lower than that of the hospitals. University-related hospitals demonstrated the highest proportion of biosimilars. We speculate that hospitals (especially university-related hospitals) may be able to reduce medical costs by introducing biosimilars, or may be more likely to be influenced by the government policy of promoting biosimilars than clinics. In contrast to hospital physicians, who follow the hospital formulary, those working in clinics can select any drug based on their preferences and that of their patients. To increase biosimilar usage, we need to further explore the factors affecting acceptance of the switch to biosimilars by healthcare providers and patients, especially at clinics.

Furthermore, the specific characteristics of the Japanese healthcare system should also be taken into consideration, in addition to the preferences of healthcare providers and patients. First, substitution between original biologics and biosimilar does not occur at pharmacy because their nonproprietary names are different. This represents an important difference with the rules that apply for non-biologic generic drugs: even if a physician prescribes a brand-name drug, the patient can choose to receive a generic drug at the pharmacy instead, unless the physician prohibits the substitution in the prescription. Further, if the patient chooses a brand-name drug, additional costs may be incurred, encouraging the use of generic drugs. Moreover, some reports have suggested that, under the “High-Cost Medical Expense Benefit (Eligibility Certificate for Ceiling-Amount Application)” system (that reimburses patients for out-of-pocket medical expenses exceeding a monthly ceiling determined based on the individual's age and income) currently being applied in Japan, switching from the original biologics to biosimilars would not always result in lower out-of-pocket costs for patients [23]. These differences in the respective systems of delivery to the patient may partly explain the differing trends observed for biologics and for non-biologic drugs. In contrast to the health policy to promote the use of non-biologic generic drugs over the two decades, that of biosimilars has just started recently. It is expected that the health policy to promote the use of biosimilars will work as well.

This study has certain limitations. First, the database comprised large and medium-sized company employees and their family members; thus, the population of this study is expected to be younger and more affluent than the average Japanese population. We confirmed that the statistics in the JMDC claims database was generally similar to that obtained from the NDB Open data, but could not conduct a more sophisticated comparison by restricting the analysis to those aged < 65 years because this information was not available in the case of the NDB Open data. Second, the present findings from Japan would be informative to, but may not be directly applicable to, other countries because biosimilar usage and switching patterns are affected by various factors such as healthcare policies and reimbursement systems. Finally, although the medical institution IDs and types of medical institutions were variable information in the JMDC claims database, we were unable to obtain other information, such as the area (region) of the medical institutions and the socioeconomic status of the area.

## Conclusions

Trends in biologics utilization varied widely between different biologics and medical institution type. Although variations according to the type of biologics (ATC classification system) did not show any clearly consistent pattern, a consistent pattern was observed when the type of medical institution was considered instead, with university-related hospitals and clinics being more and less likely, respectively, to introduce biosimilars compared to public and other types of hospital. Further research and strategies to increase the use of biosimilars in clinics may be needed.

## Supplementary Information

Below is the link to the electronic supplementary material.

Supplementary file1 (DOCX 59 KB).

## Data Availability

Due to our agreement with JMDC Inc., we are not allowed to share the data with readers. However, it is possible for them to purchase and obtain the data directly from JMDC Inc. The NDB Open data is publicly available.
