# Supplementary material for: Temporal Trends in the Prescription of Biosimilars and the Status of Switching from Original Biologics to Biosimilars at Individual and Institutional Levels in Japan
Source: Ther Innov Regul Sci. 2025 Aug 7;59(6):1276–87. doi: 10.1007/s43441-025-00850-7 (PMC12579695; doi:10.1007/s43441-025-00850-7)
Supplement: Supplementary file 1 — Supplementary file1 (DOCX 59 KB). [file 43441_2025_850_MOESM1_ESM.docx]

**Supplementary Table S1. List of product names and classifications**

| Name | ATC code | Original biologic | Biosimilar |
| --- | --- | --- | --- |
| 1. Somatropin | H01AC01 | Genotropin* | Somatropin BS |
|  |  | Growject | – |
|  |  | Saizen | – |
|  |  | Norditropin | – |
|  |  | Humatrope | – |
| 1. Erythropoietin*** | B03XA01 | Espo* | Epoetin Alfa BS  (Epoetin Kappa) |
|  |  | Epogin | – |
| 1. Filgrastim | L03AA02 | Gran* | Filgrastim BS |
| 1. Infliximab | L04AB02 | Remicade* | Infliximab BS |
| 1. Insulin glargine | A10AE04 | Lantus* | Insulin Glargine BS |
|  |  | Lantus XR | – |
| 1. Rituximab | L01FA01 | Rituxan* | Rituximab BS |
| 1. Etanercept | L04AB01 | Enbrel* | Etanercept BS |
| 1. Trastuzumab | L01FD01 | Herceptin* | Trastuzumab BS |
| 1. Agalsidase beta | A16AB04 | Fabrazyme* | Agalsidase Beta BS |
| 1. Bevacizumab | L01FG01 | Avastin* | Bevacizumab BS |
| 1. Darbepoetin alfa | B03XA02 | Nesp* | Darbepoetin Alfa BS |
|  |  |  | Darbepoetin Alfa** |
| 1. Teriparatide | H05AA02 | Forteo* | Teriparatide BS |
| 1. Insulin lispro | A10AB04  A10AC04  A10AD04 | Humalog* | Insulin Lispro BS |
|  |  | Humalog Mix | – |
|  |  | Humalog N | – |
|  |  | Lyumjev | – |
| 1. Adalimumab | L04AB04 | Humira* | Adalimumab BS |
| 1. Insulin aspart | A10AB05  A10AD05 | NovoRapid* | Insulin Aspart BS |
|  |  | NovoRapid Mix | – |
|  |  | Fiasp | – |
| 1. Ranibizumab | S01LA04 | Lucentis* | Ranibizumab BS |
| 1. Pegfilgrastim | L03AA13 | G-lasta* | Pegfilgrastim BS |

* Reference product of biosimilar.

** Authorized generic (AG) biologic drug.

***Different from other biologics, “Erythropoietin” is not a generic name but used here because multiple active ingredients exist for original biologics, including epoetin alfa (Espo) and epoetin beta (Epogin).

| Anatomical Therapeutic Chemical classification | Names and the proportion of biosimilar prescriptions (among all prescriptions of biologics) in May 2024 | | | | | | | |
| --- | --- | --- | --- | --- | --- | --- | --- | --- |
| A: ALIMENTARY TRACT AND METABOLISM | (5) Insulin glargine: 59.2%  Sensitivity analysis*: 83.0% | (9) Agalsidase beta: 25.6% | (13) Insulin lispro: 35.0%  Sensitivity analysis*: 45.8% | (15) Insulin aspart: 23.6%  Sensitivity analysis*: 28.6% |  |  |  |  |
| B: BLOOD AND BLOOD FORMING ORGANS | (2) Erythropoietin: 36.7%  Sensitivity analysis*: 49.2% | (11) Darbepoetin alfa: 86.2%  Sensitivity analysis*: 53.4% |  |  |  |  |  |  |
| H: SYSTEMIC HORMONAL PREPARATIONS, EXCL. SEX HORMONES AND INSULINS | (1) Somatropin: 13.6%  Sensitivity analysis*: 42.4% | (12) Teriparatide: 73.4% |  |  |  |  |  |  |
| L: ANTINEOPLASTIC AND IMMUNOMODULATING AGENTS | (3) Filgrastim: 92.5% | (4) Infliximab: 37.7% | (6) Rituximab: 81.8% | (7) Etanercept: 68.9% | (8) Trastuzumab: 81.6% | (10) Bevacizumab: 57.5% | (14) Adalimumab: 27.0% | (17) Pegfilgrastim: 26.0% |
| S: SENSORY ORGANS | (16) Ranibizumab: 75.5% |  |  |  |  |  |  |  |

**Supplementary Table S2. The proportion of biosimilar prescriptions (among all prescriptions of biologics) in May 2024** **according to the Anatomical Therapeutic Chemical classification**

*While all the original biologics or biosimilars available in Japan were included in the main analysis, the sensitivity analysis made the changes below (corresponding to Supplementary Table S1):

- For somatropin, we considered Genotropin vs. Somatropin BS.
- For erythropoietin, we considered Espo vs. Epoetin Alfa BS (Epoetin Kappa).
- For insulin glargine, we considered Lantus (not including Lantus XR) vs. Insulin Glargine BS.
- For darbepoetin alfa, we considered Nesp vs. Darbepoetin Alfa BS (not including Darbepoetin Alfa authorized generic).
- For insulin lispro, we considered Humalog (not including Humalog Mix and Humalog N) vs. Insulin Lispro BS.
- For insulin aspart, we considered NovoRapid (not including NovoRapid Mix) vs. Insulin Aspart BS.

**Supplementary Table S3. Comparison of number of prescriptions and proportion of biosimilars between the JMDC claims database and the NDB Open Data from April 2022 to March 2023**

| Name | JMDC claims database from April 2022 to March 2023 | | NDB Open Data from April 2022 to March 2023 | |
| --- | --- | --- | --- | --- |
|  | Total no. of prescriptions for both original biologics and biosimilars | No. of prescriptions for biosimilars (%) | Total no. of prescriptions for both original biologics and biosimilars | Total no. of prescriptions for both original biologics and biosimilars |
| 1. Somatropin | 34,931 | 2,859 (8.2) | 1087,245 | 84,386 (7.8) |
| Sensitivity analysis* | 8,943 | 2,859 (32.0) | 285,465 | 84,386 (29.6) |
| 1. Erythropoietin | 16,083 | 5,278 (32.8) | 337,188 | 121,294 (36.0) |
| Sensitivity analysis* | 12,140 | 5,278 (43.5) | 186,132 | 121,294 (65.2) |
| 1. Filgrastim | 39,343 | 36,552 (92.9) | 907,989 | 861,176 (94.8) |
| 1. Infliximab | 25,590 | 7,349 (28.7) | 937,777 | 272,924 (29.1) |
| 1. Insulin glargine | 162,849 | 91,755 (56.3) | 6070,213 | 3267,121 (53.8) |
| Sensitivity analysis* | 113,707 | 91,755 (80.7) | 4446,768 | 3267,121 (73.5) |
| 1. Rituximab | 11,134 | 7,986 (71.7) | 357,789 | 282,047 (78.8) |
| 1. Etanercept | 23,607 | 13,654 (57.8) | 1925,756 | 898,041 (46.6) |
| 1. Trastuzumab | 29,597 | 19,915 (67.3) | 918,232 | 602,321 (65.6) |
| 1. Agalsidase beta | 1,808 | 411 (22.7) | 45,713 | 8,063 (17.6) |
| (10) Bevacizumab | 37,689 | 9,426 (25.0) | 1577,373 | 425,443 (27.0) |
| (11) Darbepoetin alfa | 14,436 | 12,550 (86.9) | 1518,781 | 1198,118 (78.9) |
| Sensitivity analysis* | 4,293 | 2,407 (56.1) | 522,992 | 201,751 (38.6) |
| (12) Teriparatide | 9,071 | 5,082 (56.0) | 754,299 | 329,326 (43.7) |
| (13) Insulin lispro | 156,459 | 42,161 (26.9) | 9079,238 | 1824,060 (20.1) |
| Sensitivity analysis* | 120,711 | 42,161 (34.9) | 6552,217 | 1824,060 (27.8) |
| (14) Adalimumab | 29,496 | 3,926 (13.3) | 1090,901 | 136,087 (12.5) |
| (15) Insulin aspart | 132,168 | 12,139 (9.2) | 8663,964 | 535,289 (6.2) |
| Sensitivity analysis* | 110,409 | 12,139 (11.0) | 6693,072 | 535,289 (8.0) |
| (16) Ranibizumab | 3,464 | 603 (17.4) | 168,422 | 26,819 (15.9) |
| (17) Pegfilgrastim | 16,297 | 0 (0.0) | 306,436 | 0 (0.0) |

NDB, National Database.

*While all the original biologics or biosimilars available in Japan were included in the main analysis, the sensitivity analysis made the changes below (corresponding to Supplementary Table S1):

- For somatropin, we considered Genotropin vs. Somatropin BS.
- For erythropoietin, we considered Espo vs. Epoetin Alfa BS (Epoetin Kappa).
- For insulin glargine, we considered Lantus (not including Lantus XR) vs. Insulin Glargine BS.
- For darbepoetin alfa, we considered Nesp vs. Darbepoetin Alfa BS (not including Darbepoetin Alfa authorized generic).
- For insulin lispro, we considered Humalog (not including Humalog Mix and Humalog N) vs. Insulin Lispro BS.
- For insulin aspart, we considered NovoRapid (not including NovoRapid Mix) vs. Insulin Aspart BS.

**Supplementary Table S4. Distribution of patients using only original biologics or biosimilars or switchers during the study period**

| Drug | Main analysis from Jan, 2005 to May, 2024 | | | | | | Additional analysis restricting to patients using drugs only during the period from when each biosimilar was approved to May, 2024 | | | | | | | |
| --- | --- | --- | --- | --- | --- | --- | --- | --- | --- | --- | --- | --- | --- | --- |
|  | Switchers from original biologics to biosimilars | Patients using only biosimilars | Switchers from biosimilars to original biologics | Patients using only original biologics | Not classified* | Total no. of patients | When each biosimilar was approved | Switchers from original biologics to biosimilars | Patients using only biosimilars | Switchers from biosimilars to original biologics | Patients using only original biologics | Not classified* | Total no. of patients |  |
| (1) Somatropin | 440  (3.9%) | 734  (6.5%) | 47  (0.4%) | 10,042  (89.2%) | 1  (<0.1%) | 11,264  (100%) | Jun, 2009 | 439  (4.0%) | 734  (6.6%) | 47  (0.4%) | 9,835  (89.0%) | 1  (<0.1%) | 11,056  (100%) |  |
| Sensitivity analysis** | 83  (2.2%) | 1,132  (30.5%) | 6  (0.2%) | 2,487  (67.1%) | 1  (<0.1%) | 3,709  (100%) |  | 82  (2.3%) | 1,132  (31.2%) | 6  (0.2%) | 2,404  (66.3%) | 1  (<0.1%) | 3,625  (100%) |  |
| (2) Erythropoietin | 252  (1.2%) | 2,864  (14.2%) | 122  (0.6%) | 16,812  (83.3%) | 125  (0.6%) | 20,175  (100%) | Jan, 2010 | 234  (1.2%) | 2,864  (14.7%) | 122  (0.6%) | 16,102  (82.8%) | 125  (0.6%) | 19,447  (100%) |  |
| Sensitivity analysis** | 74  (0.5%) | 3,211  (22.4%) | 36  (0.3%) | 10,981  (76.6%) | 42  (0.3%) | 14,344  (100%) |  | 68  (0.5%) | 3,211  (23.2%) | 36  (0.3%) | 10,509  (75.8%) | 42  (0.3%) | 13,866  (100%) |  |
| (3) Filgrastim | 590  (2.3%) | 19,151  (74.4%) | 415  (1.6%) | 5,427  (21.1%) | 144  (0.6%) | 25,727  (100%) | Nov, 2012 | 558  (2.3%) | 19,151  (78.5%) | 415  (1.7%) | 4,129  (16.9%) | 144  (0.6%) | 24,397  (100%) |  |
| (4) Infliximab | 1,080  (10.7%) | 1,507  (14.9%) | 86  (0.9%) | 7,451  (73.5%) | 12  (0.1%) | 10,136  (100%) | Jul, 2014 | 918  (10.7%) | 1,507  (17.5%) | 86  (1.0%) | 6,090  (70.7%) | 12  (0.1%) | 8,613  (100%) |  |
| (5) Insulin glargine | 5,142  (8.3%) | 27,208  (43.9%) | 1,350  (2.2%) | 27,838  (44.9%) | 500  (0.8%) | 62,038  (100%) | Dec, 2014 | 3,603  (6.7%) | 27,208  (50.4%) | 1,350  (2.5%) | 21,361  (39.5%) | 500  (0.9%) | 54,022  (100%) |  |
| Sensitivity analysis** | 4,440  (8.6%) | 28,903  (56.3%) | 489  (1.0%) | 17,181  (33.4%) | 368  (0.7%) | 51,381 (100%) |  | 2,901  (6.7%) | 28,903  (66.7%) | 489  (1.1%) | 10,704  (24.7%) | 368  (0.9%) | 43,365  (100%) |  |
| (6) Rituximab | 398  (4.8%) | 3,105  (37.6%) | 138  (1.7%) | 4,552  (55.2%) | 59  (0.7%) | 8,252  (100%) | Sep, 2017 | 279  (4.5%) | 3,105  (49.5%) | 138  (2.2%) | 2,693  (42.9%) | 59  (0.9%) | 6,274  (100%) |  |
| (7) Etanercept | 1,053  (14.0%) | 2,671  (35.5%) | 128  (1.7%) | 3,649  (48.5%) | 20  (0.3%) | 7,521  (100%) | Jan, 2018 | 529  (10.5%) | 2,671  (53.0%) | 128  (2.5%) | 1,695  (33.6%) | 20  (0.4%) | 5,043  (100%) |  |
| (8) Trastuzumab | 811  (8.9%) | 2,992  (32.8%) | 45  (0.5%) | 5,275  (57.7%) | 13  (0.1%) | 9,136  (100%) | Mar, 2018 | 687  (11.1%) | 2,992  (48.3%) | 45  (0.7%) | 2,463  (39.7%) | 13  (0.2%) | 6,200  (100%) |  |
| (9) Agalsidase beta | 10  (9.8%) | 16  (15.7%) | 0  (0%) | 76  (74.5%) | 0  (0%) | 102  (100%) | Sep, 2018 | 4  (5.7%) | 16  (22.9%) | 0  (0%) | 50  (71.4%) | 0  (0%) | 70  (100%) |  |
| (10) Bevacizumab | 873  (6.1%) | 1,823  (12.8%) | 31  (0.2%) | 11,525  (80.8%) | 9  (0.1%) | 14,261  (100%) | Jun, 2019 | 747  (8.8%) | 1,823  (21.5%) | 31  (0.4%) | 5,881  (69.3%) | 9  (0.1%) | 8,491  (100%) |  |
| (11) Darbepoetin alfa | 1,542  (11.8%) | 5,942  (45.4%) | 153  (1.2%) | 5,382  (41.1%) | 64  (0.5%) | 13,083  (100%) | Sep, 2019 | 216  (3.1%) | 5,927  (85.8%) | 153  (2.2%) | 557  (8.1%) | 57  (0.8%) | 6,910  (100%) |  |
| Sensitivity analysis** | 273  (3.2%) | 1,350  (5.9%) | 16  (0.2%) | 6,839  (80.5%) | 13  (0.2%) | 8,491  (100%) |  | 65  (2.8%) | 1,350  (57.9%) | 16  (0.7%) | 889  (38.1%) | 13  (0.6%) | 2,333  (100%) |  |
| (12) Teriparatide | 205  (3.8%) | 1,236  (23.1%) | 26  (0.5%) | 3,872  (72.4%) | 6  (0.1%) | 5,345  (100%) | Sep, 2019 | 162  (5.9%) | 1,236  (45.1%) | 26  (1.0%) | 1,311  (47.8%) | 6  (0.2%) | 2,741  (100%) |  |
| (13) Insulin lispro | 2,924  (5.3%) | 7,663  (13.8%) | 679  (1.2%) | 44,033  (79.3%) | 212  (0.4%) | 55,511  (100%) | Mar, 2020 | 1,099  (3.7%) | 7,663  (26.0%) | 679  (2.3%) | 19,817  (67.2%) | 212  (0.7%) | 29,470  (100%) |  |
| Sensitivity analysis** | 2,780  (5.7%) | 8,052  (16.6%) | 483  (1.0%) | 37,059  (76.4%) | 163  (0.3%) | 48,537  (100%) |  | 1,021  (4.0%) | 8,052  (31.5%) | 483  (1.9%) | 15,875  (62.0%) | 163  (0.6%) | 25,594  (100%) |  |
| (14) Adalimumab | 438  (4.2%) | 1,066  (10.1%) | 40  (0.4%) | 9,002  (85.3%) | 12  (0.1%) | 10,558  (100%) | Jun, 2020 | 219  (4.2%) | 1,066  (20.6%) | 40  (0.8%) | 3,845  (74.2%) | 12  (0.2%) | 5,182  (100%) |  |
| (15) Insulin aspart | 2,132  (4.3%) | 2,182  (4.4%) | 91  (0.2%) | 45,392  (91.0%) | 62  (0.1%) | 49,859  (100%) | Mar, 2021 | 769  (4.6%) | 2,182  (13.1%) | 91  (0.6%) | 13,561  (81.4%) | 62  (0.4%) | 16,665  (100%) |  |
| Sensitivity analysis** | 2,090  (4.7%) | 2,241  (5.1%) | 81  (0.2%) | 39,776  (89.9%) | 55  (0.1%) | 44,243  (100%) |  | 758  (5.1%) | 2,241  (15.0%) | 81  (0.5%) | 11,837  (79.1%) | 55  (0.4%) | 14,972  (100%) |  |
| (16) Ranibizumab | 285  (3.5%) | 1,083  (13.3%) | 5  (0.1%) | 6,784  (83.1%) | 3  (<0.1%) | 8,160  (100%) | Sep, 2021 | 182  (6.0%) | 1,083  (35.5%) | 5  (0.2%) | 1,780  (58.3%) | 3  (0.1%) | 3,053  (100%) |  |
| (17) Pegfilgrastim | 252  (1.3%) | 269  (1.4%) | 13  (0.1%) | 19,365  (97.2%) | 22  (0.1%) | 19,921  (100%) | Sep, 2023 | 172  (6.7%) | 269  (10.5%) | 13  (0.5%) | 2,090  (81.5%) | 22  (0.9%) | 2,566  (100%) |  |

Note: For switchers, only the first switch was assessed and counted (i.e., there were some patients who switched twice or more times)

*The first prescription of original biologics and the first prescription of biosimilars occurred in the same month, so that the researchers could not differentiate which was started first.

**While all the original biologics or biosimilars available in Japan were included in the main analysis, the sensitivity analysis made the changes below (corresponding to Supplementary Table S1):

- For somatropin, we considered Genotropin vs. Somatropin BS.
- For erythropoietin, we considered Espo vs. Epoetin Alfa BS (Epoetin Kappa).
- For insulin glargine, we considered Lantus (not including Lantus XR) vs. Insulin Glargine BS.
- For darbepoetin alfa, we considered Nesp vs. Darbepoetin Alfa BS (not including Darbepoetin Alfa authorized generic).
- For insulin lispro, we considered Humalog (not including Humalog Mix and Humalog N) vs. Insulin Lispro BS.
- For insulin aspart, we considered NovoRapid (not including NovoRapid Mix) vs. Insulin Aspart BS.

**Supplementary Table S5. Distribution of patients using only original biologics or biosimilars or switchers during the study period after restricting to patients visiting (at least one time) medical institutions prescribing biosimilars during the study period**

| Drug | Main analysis from Jan, 2005 to May, 2024 | | | | | | Additional analysis restricting to patients using drugs only during the period from when each biosimilar was approved to May, 2024 | | | | | | | |
| --- | --- | --- | --- | --- | --- | --- | --- | --- | --- | --- | --- | --- | --- | --- |
|  | Switchers from original biologics to biosimilars | Patients using only biosimilars | Switchers from biosimilars to original biologics | Patients using only original biologics | Not classified* | Total no. of patients | When each biosimilar was approved | Switchers from original biologics to biosimilars | Patients using only biosimilars | Switchers from biosimilars to original biologics | Patients using only original biologics | Not classified* | Total no. of patients |  |
| (1) Somatropin | 440  (7.4%) | 734  (12.4%) | 47  (0.8%) | 4,692  (79.3%) | 1  (<0.1%) | 5,914  (100%) | Jun, 2009 | 439  (7.5%) | 734  (12.6%) | 47  (0.8%) | 4,615  (79.1%) | 1  (<0.1%) | 5,836  (100%) |  |
| Sensitivity analysis** | 83  (3.6%) | 1,132  (48.6%) | 6  (0.3%) | 1,108  (47.6%) | 1  (<0.1%) | 2,330  (100%) |  | 82  (3.6%) | 1,132  (49.5%) | 6  (0.3%) | 1,068  (46.7%) | 1  (<0.1%) | 2,289  (100%) |  |
| (2) Erythropoietin | 252  (3.0%) | 2,864  (33.8%) | 122  (1.4%) | 5,116  (60.3%) | 125  (1.5%) | 8,479  (100%) | Jan, 2010 | 234  (2.9%) | 2,864  (35.5%) | 122  (1.5%) | 4,735  (58.6%) | 125  (1.6%) | 8,080  (100%) |  |
| Sensitivity analysis** | 74  (1.1%) | 3,211  (45.4%) | 36  (0.5%) | 3,718  (52.5%) | 42  (0.6%) | 7,081  (100%) |  | 68  (1.0%) | 3,211  (46.9%) | 36  (0.5%) | 3,493  (51.0%) | 42  (0.6%) | 6,850  (100%) |  |
| (3) Filgrastim | 590  (2.4%) | 19,151  (76.5%) | 415  (1.7%) | 4,746  (19.0%) | 144  (0.6%) | 25,046  (100%) | Nov, 2012 | 558  (2.3%) | 19,151  (80.4%) | 415  (1.7%) | 3,540  (14.9%) | 144  (0.6%) | 23,808  (100%) |  |
| (4) Infliximab | 1,080  (15.5%) | 1,507  (21.6%) | 86  (1.2%) | 4,297  (61.5%) | 12  (0.2%) | 6,982  (100%) | Jul, 2014 | 918  (15.4%) | 1,507  (25.2%) | 86  (1.4%) | 3,459  (57.8%) | 12  (0.2%) | 5,982  (100%) |  |
| (5) Insulin glargine | 5,142  (9.2%) | 27,208  (48.5%) | 1,350  (2.4%) | 21,856  (39.0%) | 500  (0.9%) | 56,056  (100%) | Dec, 2014 | 3,603  (7.3%) | 27,208  (55.2%) | 1,350  (2.7%) | 16,596  (33.7%) | 500  (1.0%) | 49,257  (100%) |  |
| Sensitivity analysis** | 4,440  (9.4%) | 28,903  (61.3%) | 489  (1.0%) | 12,928  (27.4%) | 368  (0.8%) | 47,128 (100%) |  | 2,901  (7.2%) | 28,903  (71.6%) | 489  (1.2%) | 7,699  (19.1%) | 368  (0.9%) | 40,360  (100%) |  |
| (6) Rituximab | 398  (5.5%) | 3,105  (42.7%) | 138  (1.9%) | 3,577  (49.2%) | 59  (0.8%) | 7,277  (100%) | Sep, 2017 | 279  (5.0%) | 3,105  (55.9%) | 138  (2.5%) | 1,972  (35.5%) | 59  (1.1%) | 5,553  (100%) |  |
| (7) Etanercept | 1,053  (16.9%) | 2,671  (42.8%) | 128  (2.1%) | 2,367  (37.9%) | 20  (0.3%) | 6,239  (100%) | Jan, 2018 | 529  (12.1%) | 2,671  (61.0%) | 128  (2.9%) | 1,034  (23.6%) | 20  (0.5%) | 4,382  (100%) |  |
| (8) Trastuzumab | 811  (11.1%) | 2,992  (40.8%) | 45  (0.6%) | 3,471  (47.3%) | 13  (0.2%) | 7,332  (100%) | Mar, 2018 | 687  (13.4%) | 2,992  (58.4%) | 45  (0.9%) | 1,388  (27.1%) | 13  (0.3%) | 5,125  (100%) |  |
| (9) Agalsidase beta | 10  (22.7%) | 16  (36.4%) | 0  (0%) | 18  (40.9%) | 0  (0%) | 44  (100%) | Sep, 2018 | 4  (13.3%) | 16  (53.3%) | 0  (0%) | 10  (33.3%) | 0  (0%) | 30  (100%) |  |
| (10) Bevacizumab | 873  (9.0%) | 1,823  (18.8%) | 31  (0.3%) | 6,946  (71.7%) | 9  (0.1%) | 9,682  (100%) | Jun, 2019 | 747  (12.6%) | 1,823  (30.7%) | 31  (0.5%) | 3,326  (56.0%) | 9  (0.2%) | 5,936  (100%) |  |
| (11) Darbepoetin alfa | 1,542  (12.9%) | 5,942  (49.6%) | 153  (1.3%) | 4,286  (35.8%) | 64  (0.5%) | 11,987  (100%) | Sep, 2019 | 216  (3.3%) | 5,927  (89.9%) | 153  (2.3%) | 239  (3.6%) | 57  (0.9%) | 6,592  (100%) |  |
| Sensitivity analysis** | 273  (10.0%) | 1,350  (49.6%) | 16  (0.6%) | 1,072  (39.4%) | 13  (0.5%) | 2,724  (100%) |  | 65  (4.4%) | 1,350  (91.0%) | 16  (1.1%) | 39  (2.6%) | 13  (0.9%) | 1,483  (100%) |  |
| (12) Teriparatide | 205  (7.5%) | 1,236  (45.1%) | 26  (1.0%) | 1,266  (46.2%) | 6  (0.2%) | 2,739  (100%) | Sep, 2019 | 162  (9.0%) | 1,236  (68.7%) | 26  (1.4%) | 370  (20.6%) | 6  (0.3%) | 1,800  (100%) |  |
| (13) Insulin lispro | 2,924  (8.5%) | 7,663  (22.3%) | 679  (2.0%) | 22,887  (66.6%) | 212  (0.6%) | 34,365  (100%) | Mar, 2020 | 1,099  (5.8%) | 7,663  (40.2%) | 679  (3.6%) | 9,420  (49.4%) | 212  (1.1%) | 19,073  (100%) |  |
| Sensitivity analysis** | 2,780  (9.0%) | 8,052  (26.1%) | 483  (1.0%) | 19,387  (62.8%) | 163  (0.5%) | 30,865  (100%) |  | 1,021  (6.0%) | 8,052  (47.2%) | 483  (2.8%) | 7,334  (43.0%) | 163  (1.0%) | 17,053  (100%) |  |
| (14) Adalimumab | 438  (6.8%) | 1,066  (16.5%) | 40  (0.6%) | 4,903  (75.9%) | 12  (0.2%) | 6,459  (100%) | Jun, 2020 | 219  (6.6%) | 1,066  (32.0%) | 40  (1.2%) | 1,990  (59.8%) | 12  (0.4%) | 3,327  (100%) |  |
| (15) Insulin aspart | 2,132  (8.6%) | 2,182  (8.8%) | 91  (0.4%) | 20,429  (82.1%) | 62  (0.3%) | 24,896  (100%) | Mar, 2021 | 769  (9.0%) | 2,182  (25.5%) | 91  (1.1%) | 5,467  (63.8%) | 62  (0.7%) | 8,571  (100%) |  |
| Sensitivity analysis** | 2,090  (9.1%) | 2,241  (9.8%) | 81  (0.4%) | 18,388  (80.5%) | 55  (0.2%) | 22,855  (100%) |  | 758  (9.5%) | 2,241  (28.2%) | 81  (1.0%) | 4,821  (60.6%) | 55  (0.7%) | 7,956  (100%) |  |
| (16) Ranibizumab | 285  (5.5%) | 1,083  (20.9%) | 5  (0.1%) | 3,795  (73.4%) | 3  (0.1%) | 5,171  (100%) | Sep, 2021 | 182  (8.4%) | 1,083  (49.8%) | 5  (0.2%) | 902  (41.5%) | 3  (0.1%) | 2,175  (100%) |  |
| (17) Pegfilgrastim | 252  (4.3%) | 269  (4.6%) | 13  (0.2%) | 5,287  (90.5%) | 22  (0.4%) | 5,843  (100%) | Sep, 2023 | 172  (20.7%) | 269  (32.3%) | 13  (1.6%) | 356  (42.8%) | 22  (2.6%) | 832  (100%) |  |

Note: For switchers, only the first switch was assessed and counted (i.e., there were some patients who switched twice or more times)

*The first prescription of original biologics and the first prescription of biosimilars occurred in the same month, so that the researchers could not differentiate which was started first.

**While all the original biologics or biosimilars available in Japan were included in the main analysis, the sensitivity analysis made the changes below (corresponding to Supplementary Table S1):

- For somatropin, we considered Genotropin vs. Somatropin BS.
- For erythropoietin, we considered Espo vs. Epoetin Alfa BS (Epoetin Kappa).
- For insulin glargine, we considered Lantus (not including Lantus XR) vs. Insulin Glargine BS.
- For darbepoetin alfa, we considered Nesp vs. Darbepoetin Alfa BS (not including Darbepoetin Alfa authorized generic).
- For insulin lispro, we considered Humalog (not including Humalog Mix and Humalog N) vs. Insulin Lispro BS.
- For insulin aspart, we considered NovoRapid (not including NovoRapid Mix) vs. Insulin Aspart BS.
